# German translation, cultural adaptation and validation of the unidimensional self-efficacy scale for multiple sclerosis

**DOI:** 10.1186/s12883-021-02183-y

**Published:** 2021-04-17

**Authors:** Barbara Seebacher, Roger J. Mills, Markus Reindl, Laura Zamarian, Simone Kircher, Christian Brenneis, Rainer Ehling, Florian Deisenhammer

**Affiliations:** 1 Clinical Department of Neurology, Medical University of Innsbruck, Anichstrasse 35, 6020 Innsbruck, Austria; 2 Department of Neurology, Walton Centre NHS Foundation Trust, Liverpool, UK; 3 Clinical Department of Neurology, University Hospital of Innsbruck, Innsbruck, Austria; 4 Department of Neurology, Clinic for Rehabilitation Münster, Münster, Austria; 5 Karl Landsteiner Institut für Interdisziplinäre Forschung, Reha Zentrum Münster, Münster, Austria

**Keywords:** Multiple sclerosis, Self-efficacy, Patient reported outcome measures, Austria, Cross-cultural comparison, Validation studies

## Abstract

**Background:**

Self-efficacy concerns individuals’ beliefs in their capability to exercise control in specific situations and complete tasks successfully. In people with multiple sclerosis (PwMS), self-efficacy has been associated with physical activity levels and quality of life. As a validated German language self-efficacy scale for PwMS is missing the aims of this study were to translate the Unidimensional Self-Efficacy Scale for Multiple Sclerosis (USE-MS) into German, establish face and content validity and cultural adaptation of the German version for PwMS in Austria. A further aim was to validate the German USE-MS (USE-MS-G) in PwMS.

**Methods:**

Permission to translate and validate the USE-MS was received from the scale developers. Following guidelines for translation and validation of questionnaires and applying Bandura’s concept of self-efficacy, the USE-MS was forward-backward translated with content and face validity established. Cultural adaptation for Austria was performed using cognitive patient interviews. Reliability was assessed using Cronbach’s alpha, Person separation index and Lin’s concordance correlation coefficient. Rasch analysis was employed to assess construct validity. Comparison was made to scales for resilience, general self-efficacy, anxiety and depression, multiple sclerosis fatigue and health-related quality of life. Data were also pooled with an historic English dataset to compare the English and German language versions.

**Results:**

The translation and cultural adaptation were successfully performed in the adaptation process of the USE-MS-G. Pretesting was conducted in 30 PwMS, the validation of the final USE-MS-G involved 309 PwMS with minimal to severe disability. The USE-MS-G was found to be valid against the Rasch model when fitting scale data using a bifactor solution of two super-items. It was shown to be unidimensional, free from differential item functioning and well targeted to the study population. Excellent convergent and known-groups validity, internal consistency, person separation reliability and test-retest reliability were shown for the USE-MS-G. Pooling of the English and German datasets confirmed invariance of item difficulties between languages.

**Conclusion:**

The USE-MS-G is a robust, valid and reliable scale to assess self-efficacy in PwMS and can generate interval level data on an equivalent metric to the UK version.

**Trial registration:**

ISRCTN Registry; ISRCTN14843579; prospectively registered on 02. 01. 2019.

**Supplementary Information:**

The online version contains supplementary material available at 10.1186/s12883-021-02183-y.

## Background

Multiple Sclerosis (MS) is a chronic demyelinating disease of the central nervous system, with accumulating disability and loss in quality of life [1]. It appears relevant for people with MS (PwMS) to preserve their autonomy, despite functional limitations and an unpredictable disease course.

Self-efficacy is described as belief in one’s ability to perform relevant tasks in order to overcome challenges and to attain desirable goals. Importantly, self-efficacy is not related to people’s level of functioning or their skills but rather their judgement of what can be achieved [2]. This implies that PwMS who are confident in their ability to master challenges and reach their goals may cope with the disease more effectively. Higher levels of self-efficacy may enhance people’s motivation to be physically active as there is a strong relationship with health promoting behaviour and perceived quality of life in PwMS [3].

Several scales have been developed to assess self-efficacy in PwMS [4–6]. The Unidimensional Self-Efficacy Scale for Multiple Sclerosis (USE-MS) [5] was developed from the Liverpool Self-efficacy Scale (LSES) [6] and the Multiple Sclerosis Self-Efficacy Scale (MSSS) [4], both of which resulted from in-depth patient interviews and were underpinned by Bandura’s theory of self-efficacy. The USE-MS was the only scale to meet the stringent criteria of a Rasch model [7] used for assessing its psychometric properties in a large sample of PwMS [5]. Accordingly, the USE-MS is a valid and reliable instrument for use in clinical practice and research. So far, however, a validated German language version of the USE-MS is missing. Therefore, the main aim of this study was to translate the USE-MS into German and validate the German language version (USE-MS-G) using correlational and Rasch analysis. A further aim was to examine the scale for language invariance and to equate the English USE-MS and German USE-MS-G.

## Methods

### Study design and setting

A prospective cross-sectional translation and validation study with repeated measures and consisting of two phases, was conducted at the outpatient MS-Clinic of the Clinical Department of Neurology, Medical University of Innsbruck, Austria and Department of Neurology, Clinic for Rehabilitation Münster, Austria from 12.2.2019 to 15.06.2020. Ethical approval was received from the Ethics Committee of the Medical University of Innsbruck (reference number EK1260/2018; 13.12.2018). See Additional file 1 for a completed STROBE checklist.

### Study phases

In Phase 1, the forward-backward translation, establishment of face and content validity and cross-cultural adaptation of the pre-final USE-MS-G were performed. Validity and reliability testing of the USE-MS-G were completed in Phase 2.

### Participants

Printed information and invitations to participate in the study were displayed in the MS-Clinic, the Clinic for Rehabilitation, the Austrian MS Society patient magazine and on their website; they were also forwarded to MS support groups. In order to facilitate participation, severely disabled PwMS (Expanded Disability Status Scale (EDSS) [8] ≥8) were visited at home in order to be informed about the study. During their regular visits, PwMS were notified about the study by Clinic staff. All procedures followed the tenets of the Declaration of Helsinki and written informed consent was obtained from all participants.

A random cross-sectional cohort of patients with clinically definite MS in accordance with the McDonald’s criteria [9] version valid at the time of their diagnosis presenting any MS phenotype was recruited into this study. PwMS of any ethnicity with very good German language skills, aged ≥18 years with different levels of functioning were included (EDSS scores from 0 (no disability) to 9.0 (severe disability); the detailed study protocol can be found elsewhere [10].

The exclusion criteria were comorbidities potentially affecting subjective self-efficacy ratings (such as malignant diseases, other neurological or psychiatric disorders), a relapse of MS within 2 months prior to the study or any change in medication within 4 weeks of the study commencement. A relapse or relevant clinical worsening between test and retest required the exclusion of the participant.

### Sample size

#### Phase 1

Patients were recruited until saturation was reached, indicating that no further information could be obtained from conducting additional interviews.

#### Phase 2

According to previous recommendations, [11, 12] for the Rasch analysis, the aim was to recruit a minimum of 250 cases presenting a wide range of self-efficacy.

### Outcome measures and procedures

Demographic (gender, age) and disease specific data (disease duration, MS phenotype, disease-modifying treatment) were retrieved from patients’ files. The current EDSS was assessed by neurologists. The order of the questionnaires was randomised for each participant to minimise order effects and questionnaire data were collected twice within a 14–21-day period (test, retest).

The original self-report USE-MS has been shown to be reliable and valid for self-efficacy assessment in PwMS [5]. Scoring is achieved by summating all 12 items (items 5, 7–9 and 11 are reversed scored). The USE-MS involves a 4-point Likert scale (0 = strongly disagree to 3 = strongly agree). A higher summary score signifies stronger self-efficacy beliefs in people. In this study, the USE-MS-G was self-reported by participants. The approximate completion time is 5–10 min.

Existing validated questionnaires which came at the recommendation of either government or patient organisations [13, 14] were used to evaluate convergent construct validity of the USE-MS-G. These included the validated German language versions of the General Self-Efficacy Scale (GSE) [15], Resilience Scale (RS-13) [16], Multiple Sclerosis International Quality of Life (MusiQoL) [17], Hospital Anxiety Depression Scale (HADS) [18, 19] and the Neurological Fatigue Index Multiple Sclerosis (NFI-MS) [20].

The German GSE [21] is a 10-item self-reported 4-point Likert scale (from 1 = “not at all true” to 4 = “exactly true”). Higher GSE summary scores signify greater self-efficacy. High internal consistency, moderate concurrent validity and unidimensionality have all been shown [15].

The German RS-13 [22] is based on the 25-item Resilience Scale [23] and includes a 7-point Likert scale from which a summary score is calculated. Scores of 13–66 points, 67–72 points or 73–91 points indicate low, moderate or high resilience, respectively [16]. High internal consistency, moderate test-retest reliability and an acceptable construct have been demonstrated [16].

The German MusiQoL [24] is a 31-item self-report health-related quality of life (HRQoL) instrument. Scoring is achieved on a 5-point Likert scale ranging from 1= “never/not at all” to 5 = “always/very much” and involving reverse scoring of negatively worded items. All nine domain scores and global index are standardised on a 0–100 scale, 0 indicating the worst level of HR QoL and 100 the best. Satisfactory internal and convergent validity and acceptable reliability were shown for all MusiQol dimensions [17].

The German HADS [19] is 14-item self-report questionnaire of anxiety and depression using a 4-point Likert scale and reverse scoring of negatively worded items. Higher scores indicate higher levels of anxiety or depression. Odd numbered items are combined to score the anxiety subscale (0–21 points), even items added to generate the depression subscale (0–21 points). A good internal consistency, acceptable test-retest reliability and a two-factor structure of the scale have been demonstrated [19].

The German NFI-MS [25] is a self-report questionnaire of physical (items 1–8) and cognitive (items 9–12) MS related fatigue. A summary score is generated from items 1–7, 9 and 11–12. Scoring is performed on a 4-point Likert scale from 0 = “strongly disagree” to 3 = “strongly agree” and higher scores represent worse fatigue. Good test-retest reliability, external and internal validity [26] and acceptable responsiveness [27] have been found for the NFI-MS.

### Translation, face and content validity and cultural adaptation

In Phase 1, following guidelines for the cross-cultural adaptation of patient-reported outcomes [28, 29] and the subsequently enhanced version released by the University of Leeds, UK [30], a forward-backward translation process was conducted by 6 bilingual translators, 3 native in German, 3 in English. This comprised a synthesis of translations and consensus from an expert committee of 3 neurologists, 2 physiotherapists, a clinical neuropsychologist, a methodologist, the translators and one health-care professional diagnosed with MS. Pretesting (Test 1, T1) and face-to-face cognitive interviews regarding the questionnaire wording were carried out in male and female PwMS across the disability range. Saturation was achieved after 30 recorded interviews. Cross-cultural equivalence in conceptual areas relating to semantic, idiomatic and experiential was accomplished between the USE-MS and USE-MS-G [28, 29]. Qualitative content analysis of the verbatim interview transcriptions was performed (described in detail elsewhere [10]). During all stages of the iterative adaptation process of the USE-MS-G, consultation with the UK scale developers [5] ensured that consensus was reached.

### Statistical analyses

#### Internal (construct) validity

Scale data were fitted to the Rasch measurement model [7] in order to test the assumptions required for interval level measurement such as probabilistic ordering of item difficulties and response thresholds, local independence of items, and group invariance (for gender, age, disease duration, timepoint (test, retest), centre, and subsequently language version) as well as unidimensionality [31, 32].

Rasch analysis is now a standard tool for scale validation but details of the analysis used, including fit criteria can found in the supplementary material (Additional file 2). The main challenge to the validity of most health outcome measures is the presence of item local dependency. This can be obviated by adding dependent items together to form ‘super-items’ or ‘testlets’ [33]. Constructing two super-items and incorporating all the items into a scale, is now emerging as useful method of eliminating local dependency. This method allows for the generation of a latent estimate based on a bi-factor solution which can be tested using a (more robust) conditional test of fit [34]. Fit statistics produced when using two super-items includes the explained common variance (ECV). A value of 1 indicates that all non-error variance is contained within the latent estimate with an ECV value of > 0.9 considered sufficient to indicate that the first common factor is unidimensional [35]. Rasch Analysis was conducted using RUMM2030 software (http://www.rummlab.com.au/).

#### Reliability

According to the COSMIN taxonomy, reliability comprises the measurement properties of internal consistency, reliability, and measurement error [36]. Reliability was evaluated with the Person Separation Index (PSI, range 0–1) and Cronbach’s alpha (as a measure of internal consistency), which should be ≥0.85 for individual use or 0.70 for group use [26]. Cronbach’s alpha was also calculated in the study sample for the GSE, RS-13, MusiQol, HADS and NFI-MS.

Test–retest reliability was determined using Lin’s concordance correlation coefficient [37] (r_c_) between Test 2 (T2) and Test 3 (T3). The r_c_ (0–1; 95% CI) was used to estimate the agreement level between the test and retest USE-MS-G data and was determined using MedCalc software (https://www.medcalc.org/). The Pearson correlation coefficient was calculated as a measure of precision and a Bias correction factor, C_b_ as a measure of accuracy [37].

Scale precision was examined by the standard error of measurement (SEM) and minimum detectable change (MDC) based on a 95% confidence interval (CI) [38]. Targeting of the scale was evaluated by inspection of a Person-item distribution map representing both the self-efficacy levels of the persons and the difficulty of the items. Floor and ceiling effects were assessed as the percentage of minimum and maximum extreme scores which should not exceed 5% [39]. The standard error of measurement and minimum detectable change were calculated for the USE-MS-G using the formulas SEM = standard deviation (SD) * (√ (1-Cronbach’s alpha) [40] and MDC = 1.96 * √2 * SEM) [41], where 1.96 is z-value of the 95% CI of a true difference in scores [42].

#### Convergent and known-groups validity

Spearman’s Rank correlation analyses were performed between the USE-MS-G and other measures to determine convergent construct validity. We expected moderate (r_s_ = 0.5–0.69) to high (r_s_ ≥ 0.7 [43]) positive correlations of the USE-MS-G with the GSE, RS-13 and MusiQol and moderate to high negative correlations with the HADS and NFI-MS. In order to assess the known-groups validity, subgroups of gender (female, male), disease course (relapsing, progressive MS) and levels of disability (EDSS 0–4.0; 4.5–9.0) were compared using the Mann Whitney-U test and Independent-Samples Hodges-Lehman differences (95% CI).

We hypothesised that female and male genders would have similar levels of self-efficacy, PwMS with more severe disability would have lower scores (self-efficacy) than those with milder disability (1) and PwMS with progressive MS phenotype (secondary or primary progressive) would have lower scores than those with relapsing-remitting MS (2).

Questionnaires were checked for missing item responses and participants were asked for completion to avoid missing data. Rasch analysis does not require a complete data set but calculates an estimate from all available data [44]. Descriptive statistics and convergent/known-groups validity estimates were performed using IBM SPSS software (IBM SPSS Statistics; Version 26.0. Armonk, NY: IBM Corp.) or GraphPad Prism Version 8 (GraphPad Software, La Jolla, CA). Statistical significance was defined as two-tailed *p*-value < 0.05.

## Results

### Phase 1

The prefinal USE-MS-G resulted from a forward-backward translation procedure. The 30 interviewees (23 female, 3 male) were 26–75 years old and scored 0–8.0 on the EDSS (median 3.5). Their mean disease duration was 20.3 (SD 11.7) years. Some differences were detected between the English and Austrian cultures relating to this scale. For example, feeling ‘happy and satisfied’ in relation to the things somebody does in the day (item 2) appeared somewhat over-stated for the German speaking audience; so, the wording was changed to ‘content and satisfied’. In Austria, the statement ‘Sometimes I feel inadequate as a person because of my condition’ (item 5) seemed morally inacceptable as a concept. Therefore, ‘inadequate’ was changed to ‘limited’. Additionally, the phrase ‘[I can do anything] I set my mind to’ (item 12) implied stubbornness or an extraordinary strong willpower. This was changed to ‘intend to do’ to be more acceptable. Cognitive debriefing analysis showed some discrepancies, greatest with items 3 and 5 which were solved after a second round of interviews and consensus of the expert committee in line with the original test developers.

### Phase 2

#### Population demographics

In total, 623 eligible patients were informed about the study. A number of 309 patients agreed to participate (a response rate of 49.6%) of whom 290 twice completed the questionnaires (93.8%). Nineteen patients did not complete the study as 2 participants had a relapse of MS, 11 participants could not be reached again and 6 participants reported poor health. Population characteristics are presented in Table 1 (for disease modifying treatment, DMT definitions see [10]).

**Table 1:** Population characteristics of the validation sample

Parameters	***N*** = 309
Gender^a^	72 (23.3) males
	237 (76.7) females
Age^b^	50.2 ± 11.8 years
MS phenotype^a^	194 (62.8) relapsing-remitting
	36 (11.7) primary progressive
	79 (25.6) secondary progressive
Disease duration^b^	18.21 ± 10.80 years
EDSS^c^	3.0 (0–9)
EDSS groups^a^	205 (66.3) with an EDSS of 0–4.0
	86 (27.8) with an EDSS of 4.5–6.5
	18 (5.8) with an EDSS of 7.0–9.0
Disease modifying treatment (DMT)^a^	163 (53) patients received no DMT
	61 (19.7) received lowly effective DMT^d^
	85 (27.5) received highly effective DMT^e^

*Abbreviations*: *EDSS* Expanded Disability Status Scale

^a^Data are shown as count (percentage)

^b^Data are presented as mean ± standard deviation

^c^Data are shown as median (range)

^d^low effective DMTs: interferon-b 1a and 1b, pegylated interferon-b 1a, glatiramer acetate, dimethyl fumarate, teriflunomide, azathioprin, intravenous immunoglobulins

^e^high effective DMTs: alemtuzumab, cladribine, fingolimod, natalizumab, ocrelizumab, cyclophosphamide, mitoxantrone, rituximab

#### Rasch analysis

USE-MS-G test data and pooled UK and USE-MS-G data were fitted to the Rasch model separately. Local dependency was detected in the USE-MS-G data, albeit at low level, as well as overall misfit to the Rasch model (Table 2, analysis 1). Hence, the data were combined into two super-items consisting of all alternate items (i.e., one grouping of the even numbered items and one grouping of the odd numbered items) This resulted in excellent fit parameters, unidimensionality and freedom of differential item functioning (Table 2, analysis 2). The ECV was 0.988, indicating that only less than 1% of the total scale variance was lost by using a bifactor solution. Supplementary results from the Rasch analysis are shown in Additional file 3.

**Table 2:** Model Fit of the USE-MS-G to the Rasch model

Analysis	Item residual		Person residual		(Cond.)^a^ Chi-Square^b^		PSI^c^	Alpha	Unidimensionality^d^	
	Mean	SD	Mean	SD	Value (df)	p			% tests > 5%	95% CI
**1) 12-item scale**	0.05	2.19	−0.31	1.33	138.46 (48)	0.000	0.86	0.87	23.0	5–9.9
**2) 2 super-items**	0.15	0.88	−0.59	1.01	29.24 (22)	0.138	0.85	0.86	4.2	1.8–6.6
**Ideal values**	0.00	1.00	0.00	1.00		> 0.01*	> 0.70	> 0.70	< 5.0	LCI < 5

*Abbreviations*: *T1/2* testlet, or super-item 1/2, *Cond*. conditional, *df* degrees of freedom, *PSI* person separation index, *Alpha* Cronbach’s alpha, *SD* standard deviation, *CI* confidence interval, *LCI* lower bound of the 95% CI

^a^Conditional Chi-Square: only applicable for the super-item solution; for the item-based solution, the Chi Square is shown

^b^Chi-Square of T1: 3392 (4), *p* = 0,494; Chi-Square of T2: 1054 (4), p = 0,901. Perfect values are > 0.004* (Bonferroni adjusted)

^c^The PSI indicates the reliability and differentiation of strata

^d^Based on independent t-tests to compare person residuals which are positively and negatively loading on the first principal component

*Bonferroni adjusted and variable with number of items

The UK and USE-MS-G data (*N* = 485) were also pooled and were found to be invariant by language (English; German) indicating that person estimates from either language version of the scale could be considered as equitable on a common metric.

#### Convergent and known-groups validity

Internal consistency (Cronbach’s alpha) and person separation reliability (PSI) of the USE-MS-G are presented in Table 3, together with Cronbach’s alpha values of the MusiQoL, RS-13, GSE, HADS and NFI-MS calculated in the study sample. Convergent construct validity, Spearman’s Rank correlations (95% CI) of the USE-MG-G with the MusiQoL, RS-13, GSE, HADS and NFI-MS are also demonstrated in Table 3. There were no significant differences in self-efficacy between female (median 22, range 9–36) and male (22, 6–36) genders (*p* = 0.935, median difference 0 (95% CI − 2 to 2), but there were significant differences between relapsing (24, 10–36) and progressive (19, 6–36) MS groups (*p* < 0.001; median difference 4 (95% CI 2 to 5) and also between participants with low (24, 10–36) and high (18, 6–32) disability (*p* < 0.001; median difference 5 (95% CI 4 to 7). An r_c_ of 0.92 (95% CI 0.89 to 0.93), a Pearson phi coefficient (ρ) of 0.92 and a Bias correction factor Cb of 0.99 indicated an at least moderate test-retest reliability.

**Table 3:** Convergent validity and internal consistency of comparator scales assessed for the study sample

		Cronbach’s alpha	Correlation with USE-MS-G
**Musi-QoL**	ADL	0.93	0.723 (0.664 to 0.774)***
	PWB	0.88	0.642 (0.569 to 0.705)***
	SPT	0.75	0.535 (0.447 to 0.612)***
	RFriends	0.83	0.410 (0.310 to 0.502)***
	RFamily	0.89	0.303 (0.195 to 0.403)***
	SSL	0.89	0.311 (0.202 to 0.413)***
	Cop	0.81	0.557 (0.473 to 0.632)***
	Reject	0.80	0.616 (0.539 to 0.682)***
	RHealth	0.92	0.437 (0.339 to 0.525)***
	Index^a^	0.93	0.771 (0.719 to 0.815)***
**RS-13**	Total	0.91	0.607 (0.530 to 0.675)***
**GSE**	Total	0.91	0.537 (0.450 to 0.614)***
**HADS**	Anx	0.80	−0.451 (−0.538 to −0.354)***
	Depr	0.83	−0.751 (−0.797 to − 0.696)***
	Total	0.87	−0.679 (− 0.737 to − 0.612)***
**NFI-MS**	Phys	0.92	−0.708 (− 0.761 to − 0.646)***
	Cogn	0.85	− 0.519 (− 0.598 to − 0.429)***
	Sum	0.93	−0.682 (− 0.739 to − 0.615)***

*Abbreviations*: *Musi-QoL* Multiple Sclerosis International Quality of Life questionnaire, *ADL* activities of daily living, *PWB* psychological wellbeing, *SPT* symptoms, *RFriends* relationships with friends, *RFamily* relationships with family, *SSL* sentimental and sexual life, *Cop* coping, *Reject* rejection, *RHealth* relationship with healthcare system, *RS-13* Resilience Scale-13, *GSE* General Self-Efficacy Scale, *HADS* Hospital Anxiety Depression Scale, *Anx* anxiety, *Depr* depression, *NFI-MS* Neurological Fatigue Index for MS

^a^The MusiQoL index is computed only if all dimension scores are computed (non-missing)

Spearman’s correlation coefficients are shown with 95% confidence intervals; ***correlation is significant at the < 0.001 level (2-tailed, *p*-values corrected for 18 comparisons)

A standard error of measurement (SEM) of 0.439 and a minimum detectable change (MDC) of 4.56 points were found, as measured on the original scale range of 0–36 points. That is, the smallest amount of change beyond the measurement error, expressed as MDC percentage, would be 12.7%. No floor and a 1% ceiling effect (3/309 persons) were observed and the targeting of the scale was considered good for this particular study population (Additional file 3).

#### Scoring instructions

Scoring is achieved by creating a summary score for all 12 items. Given fit to the Rasch model, a transformation of the raw score to interval scaling is available (Table 4).

**Table 4:** Transformation of raw score to interval scale latent estimate for the USE-MS-G

Raw score	Interval estimate	Raw score	Interval estimate
0	0.00	19	18.88
1	2.55	20	19.81
2	4.20	21	20.73
3	5.28	22	21.63
4	6.14	23	22.51
5	6.88	24	23.36
6	7.59	25	24.19
7	8.29	26	24.99
8	9.02	27	25.75
9	9.79	28	26.49
10	10.60	29	27.22
11	11.45	30	27.94
12	12.34	31	28.69
13	13.25	32	29.50
14	14.18	33	30.43
15	15.12	34	31.61
16	16.06	35	33.37
17	17.00	36	36.00
18	17.95		

The transformation remains valid provided there are no missing data

## Discussion

The purpose of this study was to translate, cross-culturally adapt the prefinal German USE-MS-G to Austria and validate the final USE-MS-G in PwMS across a wide range of disability. Forward-backward translation and pretesting according to guidelines are critical procedures in enabling and ensuring a high quality and reliable cross-cultural adaptation of a scale, as it is expected to better reflect the latent trait under investigation [29]. In the development studies of the MS-specific self-efficacy scales from which the USE-MS items were derived, qualitative interviews on PwMS’ experiences were used, where Bandura’s theory of self-efficacy underpinned the scales’ conceptual framework [5]. In agreement with the scale developers, we regarded it crucial to involve patients and learn from their feedback. Therefore, PwMS with different levels of disability were selected for Phase 1 of the study to gain insight into their perceptions of the prefinal USE-MS-G wording. However, even if great care is taken with this process, psychometric properties comparable to those of the original scale are not guaranteed. Therefore, fit of the German USE-MS to the Rasch model was examined in a well-powered sample of PwMS, representative of the MS population at large [45] and is comparable to the original validation study [5].

Correlation with comparator measures showed moderate to strong positive correlations with the RS-13, GSE and MusiQoL and hence corroborated the hypotheses of this study. The moderate relationship with the generic GSE stressed the importance of a disease-specific German language self-efficacy scale for PwMS. Similarly, moderate correlations of the English USE-MS with generic self-efficacy and resilience scales were also observed in the English validation study [5]. Symptoms of a chronic disease like MS suggest that affected people may hold divergent beliefs regarding their ability to perform relevant tasks and reach desired goals from those of healthy people. Further analyses showed moderate to strong negative correlations with the HADS and the NFI-MS, confirming the convergent construct being assessed. Supporting our hypotheses, females reported similar self-efficacy levels as males however, PwMS with a relapsing MS phenotype reported significantly higher self-efficacy than those with progressive MS. Likewise, USE-MS-G scores were significantly higher in participants with low disability. Our findings were in line with a previous study that used the MSSS for assessing self-efficacy [46]. Results from both studies suggest that people with severe and progressive MS may benefit from specific self-efficacy enhancing strategies. The USE-MS-G showed excellent reliability with a Cronbach’s alpha of 0.86, as compared to 0.83 in the English USE-MS study [5]. Furthermore, we observed a moderate to good test-retest reliability (r_c_ = 0.92), indicating that similar levels of self-efficacy can be expected within a short period of time from the same participants if their competencies and health status remain consistent.

The USE-MS-G demonstrated good psychometric properties with fit to the Rasch model, proven unidimensionality, and good targeting when used in a typical MS outpatient population. Within Rasch analysis, invariance of the USE-MS-G was shown for groups of different age, gender, disease duration, timepoint and centres. Invariance was also found for the English and German language versions of the USE-MS, indicating their equality. The explained common variance of the two testlets was 0.988, indicating that only less than 1% of the total scale variance was lost by using a bifactor solution. This was in agreement with the original USE-MS validation study, where an ECV of 0.96 was found [5]. Our study sample was representative to the MS population at large, with a similar female-to-male ratio, levels of disability and types of disease course when compared to a nationwide Austrian study [47]. A potential limitation of this study could be that the cultural adaptation was performed only for Austria. However, cultural differences within Germany may be similarly large than between Germany or Switzerland and Austria.

The nomogram (Table 4) can be used to generate interval level estimates of self-efficacy, suitable for parametric analysis. The minimum detectable change was 12.7% i.e., change scores of less than 4.6 points are lower than the measurement error of the scale. Item functioning of the USE-MS-G was found to be equivalent to the English version and therefore direct comparison or meta-analysis of data generated across the two countries is valid.

The English USE-MS is, to our knowledge, the most rigorously developed and tested scale for assessing self-efficacy in PwMS. The German USE-MS-G scale is now available for use in clinical and research practice as its validity and reliability has been demonstrated in this study. The USE-MS-G is easy to use and available free of charge by contacting the study authors. Assessing self-efficacy may be useful to enable an individualised and comprehensive treatment in PwMS.

## Conclusions

The USE-MS-G is a robust, valid and reliable scale to assess self-efficacy in PwMS. The translation and cross-cultural adaptation to Austria were performed according to international guidelines, relying on qualitative patient interviews to ensure the comprehensibility of the wording of questions. Fit to the Rasch model given, a transformation table from ordinal to interval scores is available for use clinical practice and research.

## Supplementary Information

**Additional file 1.** STROBE checklist.

**Additional file 2.** Supplementary methods.

**Additional file 3.** Supplementary results from Rasch analysis.

## Data Availability

The dataset(s) supporting the conclusions of this article (are) included within the article (and its additional file(s)). All data described in the manuscript, including all relevant raw data, are freely available to reviewers and any scientist wishing to use them for non-commercial purposes, without breaching participant confidentiality on reasonable request (barbara.seebacher@i-med.ac.at).

## Abbreviations

Cb: Bias correcting factor
χ^2^: Chi square test
CI: Confidence interval
DIF: Differential item functioning
DMT: Disease modifying treatment
ECV: Explained common variance
EDSS: Expanded Disability Status Scale
GSE: General Self-Efficacy Scale
HADS: Hospital Anxiety Depression Scale
LSES: Liverpool Self-efficacy Scale
MDC: Minimum detectable change
MSSS: Multiple Sclerosis Self-Efficacy Scale
MS: Multiple sclerosis
MusiQoL: Multiple Sclerosis International Quality of Life
NFI-MS: Neurological Fatigue Index Multiple Sclerosis
ρ: Pearson phi coefficient
PSI: Person Separation Index
PwMS: People with multiple sclerosis
r_c_: Lin’s concordance correlation coefficient
RS-13: Resilience Scale
SD: Standard deviation
SEM: Standard error of measurement
T1: Test 1 (Phase 1)
T2: Test 2 (rest in Phase 2)
T3: Test 3 (retest in Phase 2)
USE-MS: Unidimensional Self-efficacy Scale for Multiple Sclerosis
USE-MS-G: German version of the Unidimensional Self-efficacy Scale for Multiple Sclerosis
