# Supplementary material for: German translation, cultural adaptation and validation of the unidimensional self-efficacy scale for multiple sclerosis
Source: BMC Neurol. 2021 Apr 17;21:163. doi: 10.1186/s12883-021-02183-y (PMC8052731; doi:10.1186/s12883-021-02183-y)
Supplement: Supplementary file 2 — Additional file 2. Supplementary methods. [file 12883_2021_2183_MOESM2_ESM.docx]

**Additional File 2** Supplementary methods

Rasch analysis

Rasch analysis uses the mathematical Rasch model to assess whether a summary score for a scale can be calculated with confidence [1]. Internal construct validity of the USE-MS-G was determined by examining the deviations from model expectations, i.e. the way in which persons are expected to interact with test items to produce linear measurement [1]. The model expects that the probability of a person providing a certain answer to an item is a function of the difference between the person ‘ability’ (perceived self-efficacy) and the item ‘difficulty’. This is checked visually by inspection of item characteristic curves and numerically by the analysis of variance (ANOVA) fit statistics (uniform DIF; non-uniform DIF [2]). The USE-MS-G contains 4 response categories and hence, the polytomous Rasch model was chosen for the current study [3].

Using different chi square (χ^2^) fit statistics, USE-MS-G data were tested against the model expectations of unidimensionality. That is, the ‘ability’ and ‘difficulty’ are required to relate to the same construct of self-efficacy [3]. Using a principal component analysis (PCA) of the residuals (i.e., after the Rasch factor was removed), positive and negative loadings on the principal component were distinguished, creating two subsets and separate person estimates. Independent t-tests examined significant differences [4]. Unidimensionality of a scale is confirmed if significant t-test results outside the 95% CI do not exceed 5% or if the lower bound of the binominal CI overlaps 5% [5, 6]. Perfect values for the different fit statistics and unidimensionality are provided in Table 2. Using a residual item correlation matrix between all items the expectation of local independence was examined. Item residuals represent the difference between an item’s expected and observed values, divided by its standard deviation for standardisation. Residual correlations of +0.2 above the mean correlation of the total matrix indicate local dependence [7]. This denotes a confounding factor inducing an association between items, or multidimensionality [8, 9]. In the presence of item-dependency, two “super-items” can be created and compared with each other running a robust conditional chi-square test of fit [8]. This means that items are allocated alternately to each super-item, also called testlet or item-bundle [8, 10, 11]. Based on the assumption of a single summary score, the allocation of alternate items is expected to show an almost perfect correlation between the two super-items and absorb most or all of the local dependency within the item set [8]. The two super-items are scored polytomously and the unrestricted or partial credit model [12] is applied to them to avoid both overestimation of precision and biased estimation for item difficulty and discrimination parameters [13-15]. This approach has been shown to be suitable if local dependence levels between items within a super-item are low and the majority of scale items are independent [16]. The proportion of common to total variance retained in a testlet solution corresponds to the explained common variance (ECV) [17]. For a unidimensional scale, the ECV should be >0.9 [8], indicating that >90% of the variance is common and retained in the latent estimate [18].

The property of invariance means that all participants recognise the difficulty in identical items regardless of their self-efficacy [19]. If certain groups of participants respond differently to items, e.g. males and females, the assumption of invariance is violated, called differential item functioning (DIF) [19]. The USE-MS-G and UK data (N=485) were pooled and tested for invariance by language (English; German) to equate the language versions. Every item was examined for absence of DIF by gender (male; female), age (quartile groups), disease duration (quartile groups), timepoint (test; retest) and centre (Innsbruck; Münster). Bonferroni adjustment was performed wherever appropriate for the number of tests undertaken. If model fit was shown, a transformation table was created displaying the raw and interval scores. The distances between scores on an interval level of measurement are equal.

**References**

1. Rasch G: Probabilistic Models for Some Intelligence and Attainment Tests. Chicago: University of Chicago Press; 1980.

2. Andrich D: Rasch Models for Measurement Beverly Hills: SAGE; 1988.

3. Tennant A, Conaghan PG: The Rasch measurement model in rheumatology: what is it and why use it? When should it be applied, and what should one look for in a Rasch paper? *Arthritis and rheumatism* 2007, 57(8):1358-1362.

4. Smith EV, Jr.: Detecting and evaluating the impact of multidimensionality using item fit statistics and principal component analysis of residuals. *Journal of applied measurement* 2002, 3(2):205-231.

5. Tennant A, Pallant JF: Unidimensionality matters! (a tale of two Smiths?). *Rasch Measurement Transactions* 2006, 20:1048-1051.

6. Kersten P, White PJ, Tennant A: Is the pain visual analogue scale linear and responsive to change? An exploration using Rasch analysis. *PloS one* 2014, 9(6):e99485.

7. Christensen KB, Makransky G, Horton M: Critical Values for Yen's Q3: Identification of Local Dependence in the Rasch Model Using Residual Correlations. *Appl Psychol Meas* 2017, 41(3):178-194.

8. Pomeroy IM, Tennant A, Mills RJ, Young CA, Group TOS: The WHOQOL-BREF: a modern psychometric evaluation of its internal construct validity in people with multiple sclerosis. *Quality of Life Research* 2020, 29(7):1961-1972.

9. Andrich D: A latent trait model for items with response dependencies: Implications for test construction and analysis. In: *Test design.* edn. Edited by Embretson SE. New York: Academic Press; 1985: 245-275.

10. Wilson M, Adams RJ: Rasch models for item bundles. *Psychometrika* 1995, 60(2):181-198.

11. Rosenbaum PR: Item bundles. *Psychometrika* 1988, 53:349-359.

12. Masters G: Rasch model for partial credit scoring. *Psychometrika* 1982, 47:149–174.

13. Wang W-C, Wilson M: The Rasch testlet model. *Applied Psychological Measurement* 2005, 29(2):126-149.

14. Wainer H, Kiely GL: Item clusters and computerized adaptive testing: A case for testlets. *Journal of Educational Measurement* 1987, 24:185-201.

15. Wilson M: Detecting and interpreting local item dependence using a family of Rasch models. *Applied Psychological Measurement* 1988, 12:353-364.

16. Wainer H: Precision and differential item functioning on a testlet-based test: The 1991 Law School Admissions Test as an example. *Applied Measurement in Education* 1995, 8(2):157-186.

17. Andrich D: Components of variance of scales with a bifactor subscale structure from two calculations of α. *Educational Measurement: Issues and Practice* 2016, 35(4):25–30

18. Rodriguez A, Reise SP, Haviland MG: Evaluating bifactor models: Calculating and interpreting statistical indices. *Psychol Methods* 2016, 21(2):137-150.

19. Hagquist C, Andrich D: Recent advances in analysis of differential item functioning in health research using the Rasch model. *Health and quality of life outcomes* 2017, 15(1):181.
