# Supplementary material for: German translation, cultural adaptation and validation of the unidimensional self-efficacy scale for multiple sclerosis
Source: BMC Neurol. 2021 Apr 17;21:163. doi: 10.1186/s12883-021-02183-y (PMC8052731; doi:10.1186/s12883-021-02183-y)
Supplement: Supplementary file 3 — Additional file 3. Supplementary results from Rasch analysis. [file 12883_2021_2183_MOESM3_ESM.pdf]

Additional File 3 Supplementary results from Rasch analysis

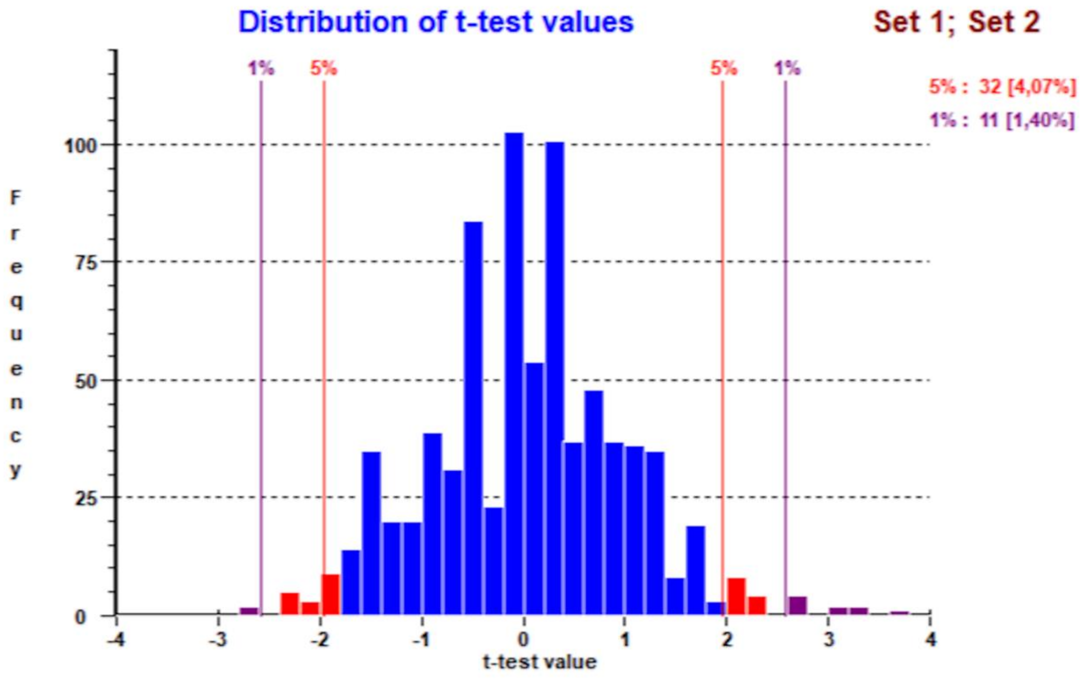

Supplementary Figure 1 Equating t-test distribution plot

This figure shows that a majority of the respondents scored similarly on the two subsets of items (person residuals which are positively and negatively loading on the first principal component). On the 5% level, 32 persons (4.07%) scored significantly different and on the 1% level, 11 persons (1.40%) showed significantly different responses.

A

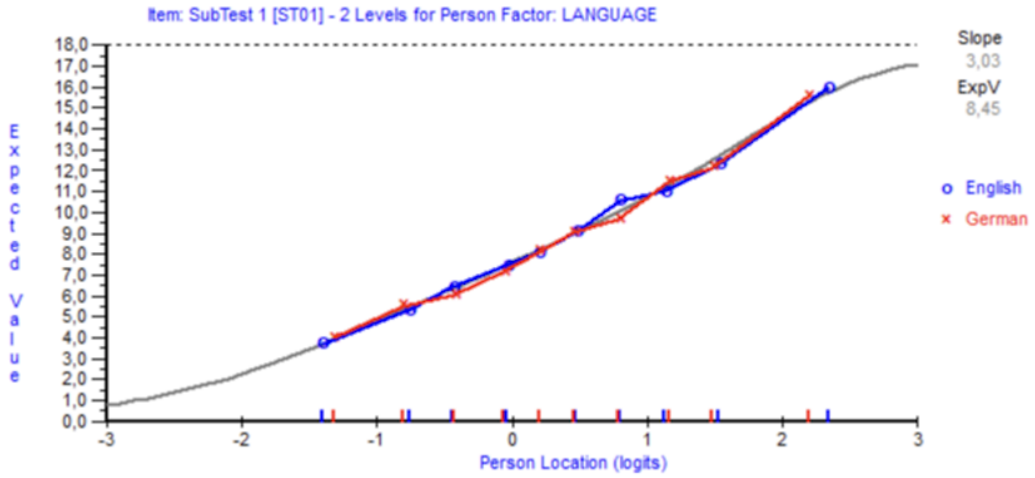

B

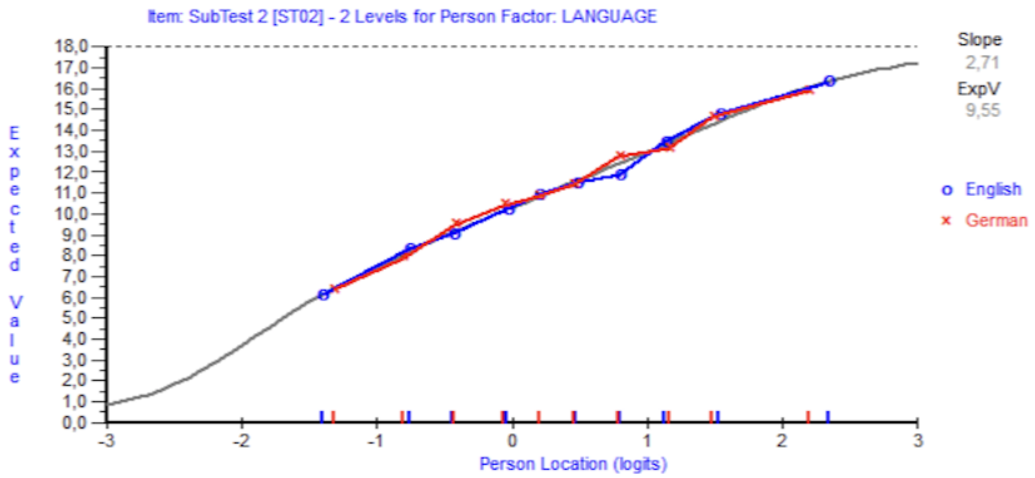

Supplementary Figure 2 Differential item functioning analyses (DIF) by language analyses results for the pooled UK and Austrian datasets

A Subtest 1 works the same way for English and German speaking participants (no DIF by language)

B Subtest 2 works the same way for English and German speaking participants (no DIF by language)

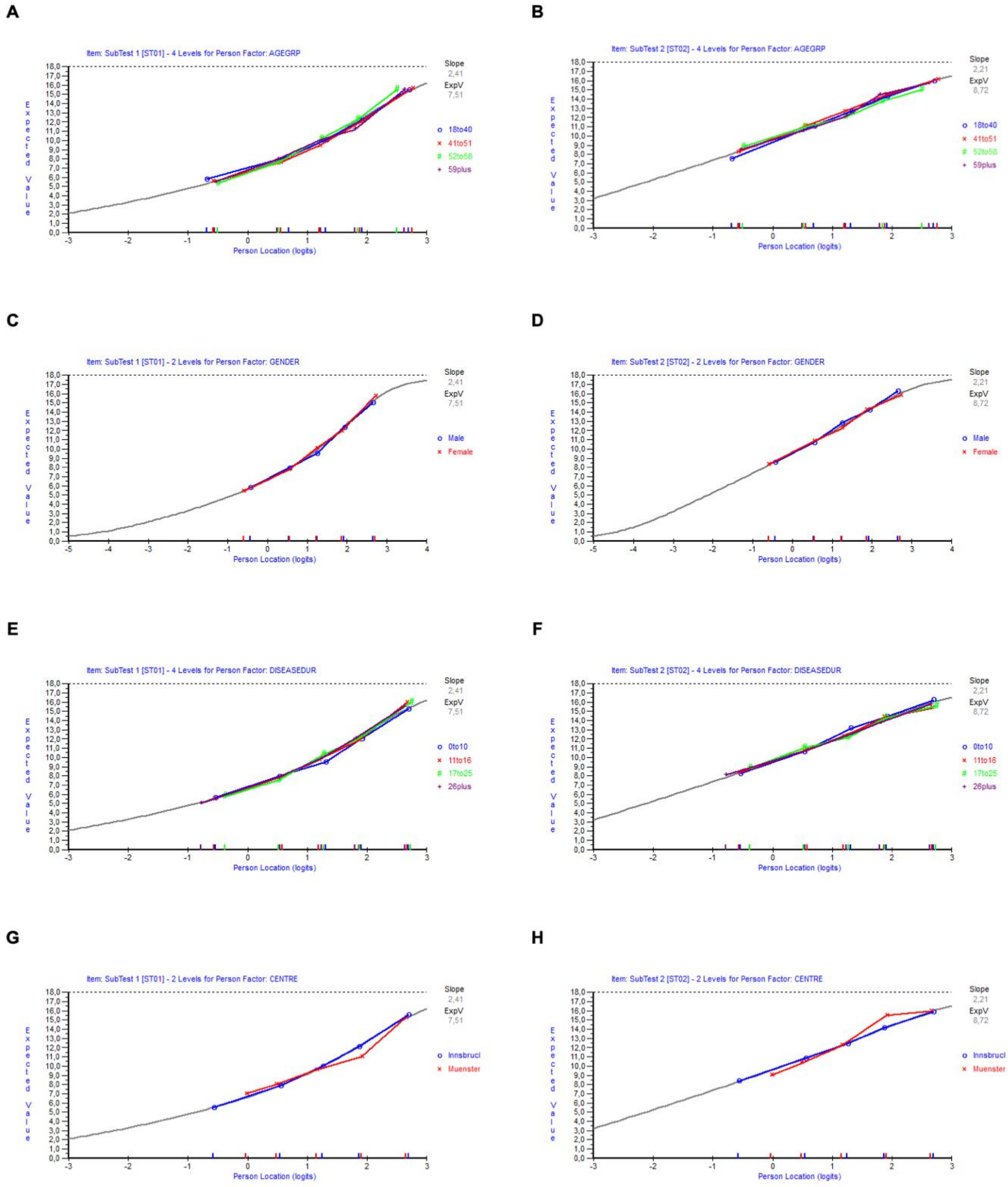

Supplementary Figure 3 Differential item functioning analyses (DIF) by age, gender, disease duration and study centre results for the Austrian dataset

The curved line (item characteristic curve) represents the expected response category endorsement at different levels of self-efficacy for the super-items. Plotted coloured lines represent the observed responses by participants at different age groups, gender, disease

duration and centre. Coloured dots on the plotted lines are the five class intervals persons were divided into according to their perceived self-efficacy levels.

A Subtest 1 works the same way for participants at different age groups (no DIF by age group)

B Subtest 2 works the same way for participants at different age groups (no DIF by age group)

C Subtest 1 works the same way for males and females (no DIF by gender)

D Subtest 2 works the same way for males and females (no DIF by gender)

E Subtest 1 works the same way for participants in different disease duration groups (no DIF by disease duration)

F Subtest 2 works the same way for participants in different disease duration groups (no DIF by disease duration)

G Subtest 1 works the same way for participants at different centres although participants from centre Innsbruck seem to have slightly higher self-efficacy (no DIF by centre shown by the ANOVA)

H Subtest 2 works the same way for participants at different centre although participants from centre Münster seem to have slightly higher self-efficacy (no DIF by centre shown by the ANOVA). In any case, the minor differences seem to cancel out at the test level

A

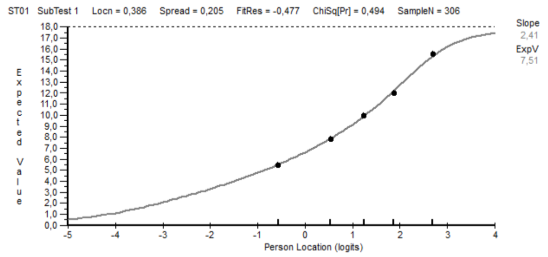

B

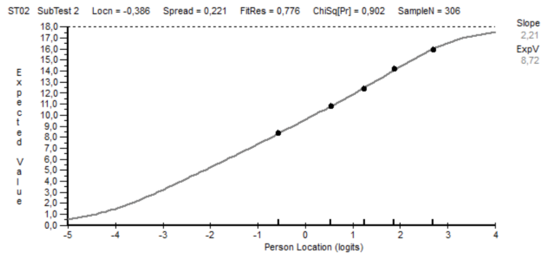

Supplementary Figure 4 Item characteristic curves

Black dots represent observed scores for the five class intervals of the persons at the different ability levels of self-efficacy. The curved line represents the expected response category endorsement at various levels of self-efficacy for the super-items. The two item characteristic curves (ICCs) indicate classic fit as the observed and expected scores match.

A ICC for subtest 1

B ICC for subtest 2

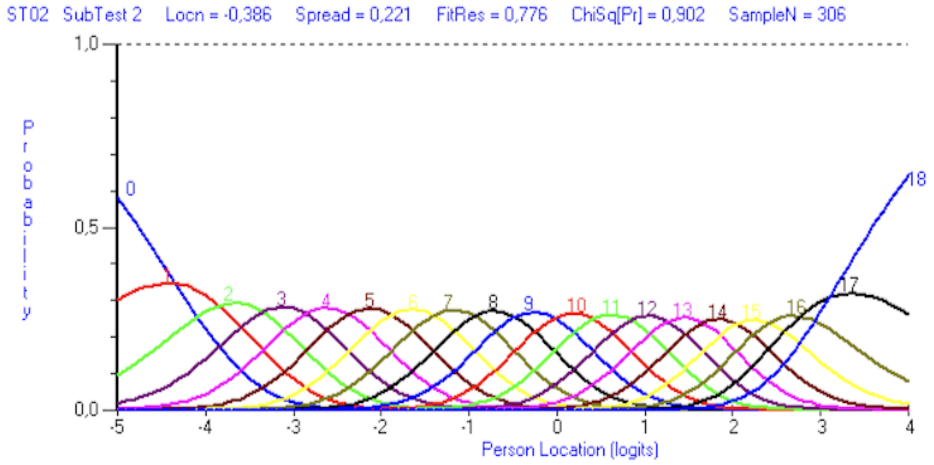

Supplementary Figure 5 Category probability curve for subtest 2

The category probability curve shows 19 ordered categories, where higher numbers represent higher levels of self-efficacy.

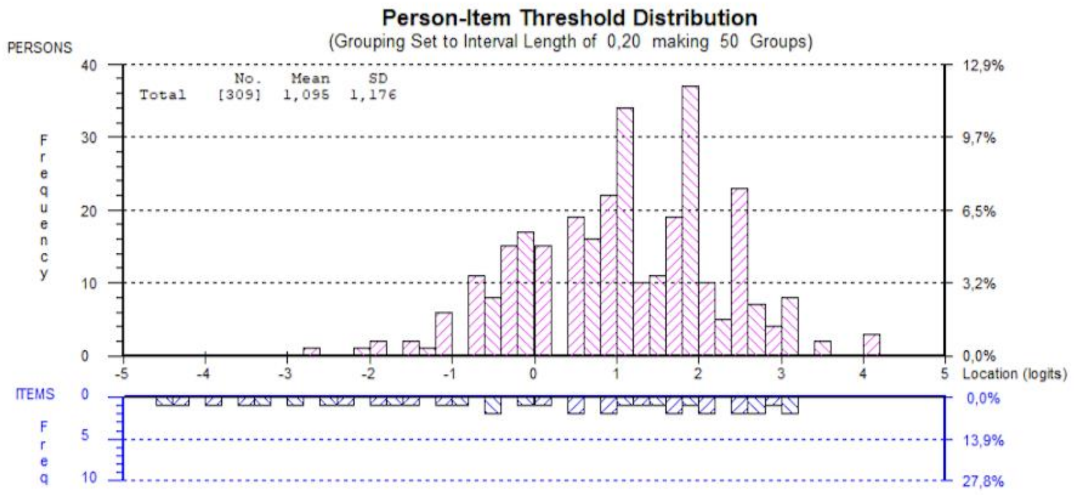

Supplementary Figure 6 Person-item threshold distribution map

Targeting of the scale is shown by the person-item threshold distribution map. Histograms on top of the plot represent the self-efficacy levels of the persons whereas those at the bottom show the difficulty of the items. On the x-axis, a logit scale is shown where the mean difficulty is set to 0 by default. On the y-axis of the top histogram, the probability of endorsing a standardised score is presented. The y-axis of the bottom histogram displays the probability of responding to a given score for a particular item.
